# Chest X‐Ray Comparison Between Drug‐Resistant and Drug‐Sensitive Pulmonary Tuberculosis in Children

**DOI:** 10.1111/crj.70010

**Published:** 2024-09-25

**Authors:** Saffanah Az Zuhriyyah, Harry Galuh Nugraha, Djatnika Setiabudi, Prayudi Santoso, Heda Melinda Nataprawira

**Affiliations:** ^1^ Faculty of Medicine Universitas Padjadjaran West Java Indonesia; ^2^ Department of Radiology, Faculty of Medicine Universitas Padjadjaran – Dr. Hasan Sadikin Hospital Bandung West Java Indonesia; ^3^ Department of Child Health, Faculty of Medicine Universitas Padjadjaran – Dr. Hasan Sadikin Hospital Bandung West Java Indonesia; ^4^ Department of Internal Medicine, Faculty of Medicine Universitas Padjadjaran – Dr. Hasan Sadikin Hospital Bandung West Java Indonesia

**Keywords:** chest X‐ray, children, pulmonary drug‐resistant, tuberculosis

## Abstract

**Introduction:**

Chest X‐ray (CXR) remains one of the tools used in diagnosing tuberculosis (TB). However, few studies about such tools exist, specifically in children in Indonesia. We aim to investigate and compare the CXR findings of children with pulmonary drug‐resistant TB (DR‐TB) and drug‐sensitive TB (DS‐TB) that could help in the evaluation and management of TB cases in children.

**Methods:**

Retrospective analysis with cross‐sectional approach was conducted in children (<18 years old) diagnosed with pulmonary DR‐TB and DS‐TB from January 2018 to December 2021. Documented data were collected from the Paediatric Respirology Registry and Tuberculosis Information System at Dr. Hasan Sadikin General Hospital Bandung. Characteristics of children, CXR findings, and TB severity were assessed and compared using the chi‐square and Fisher's exact tests with significance levels set at *p* value <0.05.

**Results:**

Sixty‐nine children (DR‐TB 31 children vs. DS‐TB 38 children) were assessed. Of the 31 children with DR‐TB, 65% were classified as multidrug‐resistant TB (MDR‐TB), followed by rifampicin‐resistant TB (RR‐TB), pre‐extensively drug‐resistant TB (pre‐XDR‐TB), and extensively drug‐resistant TB (XDR‐TB). The most common CXR findings in DR‐TB are consolidation (68%), fibrosis (42%), and cavity (29%), whereas in DS‐TB, it is pleura effusion (37%). Severe TB accounts for 50% of DR‐TB (*p* = 0.008).

**Conclusions:**

Consolidation, fibrosis, cavities, and findings of severe TB are most common in DR‐TB. Pleural effusion is the most common in DS‐TB. These findings have the potential to be considered in further examination of children with pulmonary DR‐TB and DS‐TB; hence, more extensive studies are needed to confirm these results.

## Graphical abstract

A chest X‐ray of a 16‐year‐old girl with pulmonary drug‐resistant tuberculosis (DR‐TB) is presented. Multiple cavities, fibrosis, and consolidation were seen, which are also the main findings commonly seen in children with DR‐TB in our study compared with children with pulmonary drug‐sensitive tuberculosis (DS‐TB).

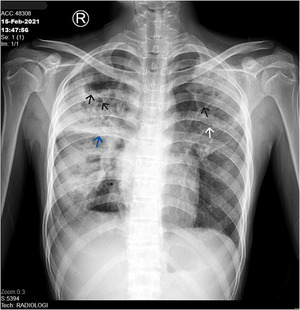

## 1 Introduction

Chest X‐ray (CXR) remains one of the tools used in diagnosing tuberculosis (TB), which locates the lesion and shows the impact of TB on the lungs [1]. Suspected TB in children should be confirmed by a CXR as there may be difficulties in specimen collection and the result of bacteriological evaluation is often negative [2, 3]. Nonetheless, regardless of its role in supporting the diagnosis, there are only a few reports of CXR findings in children with drug‐resistant TB (DR‐TB) in comparison to drug‐sensitive TB (DS‐TB), especially in Indonesia. Research conducted in Thailand reported more atelectasis and lobar consolidation in DR‐TB than DS‐TB [4]. A study of pulmonary DR‐TB in South Africa found consolidation as the most common lesion in DR‐TB, followed by lymphadenopathy, bronchopneumonic opacification, and a cavity [5].

Therefore, this study aims to investigate and compare CXR findings in children with pulmonary DR‐TB and pulmonary DS‐TB in order to establish radiological findings that could help in the evaluation and management of TB cases in children.

## 2 Materials and Methods

### 2.1 Study Design

This retrospective study was conducted to achieve the comparison of CXR in children (<18 years old) diagnosed with pulmonary DR‐TB and pulmonary DS‐TB in Dr. Hasan Sadikin General Hospital Bandung in the period of January 1, 2018, to December 31, 2021. The data were collected from the Paediatric Respirology Registry for both diagnoses and Tuberculosis Information System from the MDR‐TB clinic at Dr. Hasan Sadikin General Hospital, a tertiary hospital in Bandung, West Java.

### 2.2 Study Population

Samples were calculated using the unpaired categorical analytical research formula, resulting in the minimum sample needed being 34 children each with pulmonary DR‐TB and DS‐TB [6].

$$n_{1}=n_{2}={\left(\frac{Z_{\alpha }\;\sqrt{2\mathrm{PQ}}+Z_{\beta }\;\sqrt{P_{1}Q_{1}+P_{2}Q_{2}}\;}{P_{1}-P_{2}}\right)}^{2}$$

where Z_α_ = standard normal deviate for the two‐tailed test based on an alpha level of 5% = 1.96, Z_β_ = standard normal deviate of the beta level of 20% = 0.842, P_2_ = known proportion of one group based on a previous study = known proportion of DR‐TB children with pleural effusion CXR finding from Lapphra et al. [4] = 0.0391, P_1_ − P_2_ = difference in the minimum proportion that is considered significant = 0.25; therefore,

Q_2_ = 1 − P_2_ = 0.9609

P_1_ = proportion of a group based on the researcher's judgment = 0.2891

Q_1_ = 1 − P_1_ = 0.7109

P = total proportion = (P_1_ + P_2_)/2 = 0.1641

Q = 1 − P= 0.8359

So

$$n_{1}=n_{2}={\left(\frac{1,96\;\sqrt{2\times 0,1641\times 0,8359}+0,842\;\sqrt{0,2891\times 0,7109+0,0391\times 0,9609}\;}{0,25}\right)}^{2}$$

=33,258~34children in each group.

A total of 265 medical records of children diagnosed with TB were collected by consecutive sampling, consisting of 224 children with pulmonary TB and 41 children with extrapulmonary TB. The inclusion criteria were children <18 years old diagnosed with pulmonary TB, either DR‐TB or DS‐TB, at Dr. Hasan Sadikin General Hospital Bandung, who has CXR imaging. The exclusion criteria were lower respiratory tract non‐tuberculosis infection and incomplete medical records.

Of the 224 children with pulmonary TB, 73 children also diagnosed with non‐TB infections were excluded because of the probability of obscuring the actual cause in the existing CXR image. Another 52 children were excluded because of incomplete medical records, such as incompatible CXR interpretation to the variable, the unclear status of the time TB was first diagnosed, and incomplete TB work‐up. Thirty children who were first diagnosed outside Dr. Hasan Sadikin General Hospital Bandung were also excluded. Finally, 69 children met the inclusion criteria, of which, 31 children were diagnosed with DR‐TB and 38 children with DS‐TB.

### 2.3 Data Collection and Assessment

TB diagnosis was classified into two groups based on the Guidelines for Management of DR‐TB in Indonesia: clinically diagnosed TB and bacteriologically confirmed TB [2]. Clinically diagnosed TB considers the patient's contact history, medical history, and radiological examination, whereas bacteriologically confirmed TB involves bacteriological confirmation by an acid‐fast bacilli (AFB) smear, mycobacterial culture and identification, or DNA amplification test using X‐pert MTB/RIF. Children whose X‐pert MTB/RIF positive MTB and rifampicin sensitive were classified as DS‐TB. Children without bacteriologically confirmed diagnosis who is in stable condition and TB diagnosis supported by other examination (CXR and tuberculin test) were classified in the same group. Conversely, children with positive for MTB and rifampicin resistant were classified as DR‐TB and must undergo culture‐based phenotypic drug susceptibility testing (DST) and line probe assay (LPA) to determine the resistance category:

1. Rifampicin‐resistant TB (RR‐TB), described as tuberculosis resistant to rifampicin, with or without resistance to other anti‐tuberculosis drug [7].
2. Multidrug‐resistant TB (MDR‐TB), described as tuberculosis resistant to both isoniazid and rifampicin with or without resistant to other anti‐tuberculosis drug [7].
3. Pre‐extensively drug‐resistant TB (pre‐XDR‐TB), described as MDR‐TB that is also resistant to one of the fluoroquinolone class of drugs, including levofloxacin and moxifloxacin [8].
4. Extensively drug‐resistant TB (XDR‐TB), described as MDR‐TB that is also resistant to one of the fluoroquinolone class of drugs and at least one additional Group A drug (bedaquiline and/or linezolid) [8].

Data were also collected regarding the drug treatment status, described as the use of first‐line anti‐TB drug regimens when the CXR imaging occurred, and drug treatment history, described as previous use of first‐line anti‐TB drugs. The HIV status was based on the anti‐HIV test result reported in their medical history when diagnosed with TB.

CXR findings were based on expertise or readings by a pediatric radiology consultant in the children's medical record with anteroposterior or posteroanterior projections and lateral if present. CXR expertise was classified into variables: normal, hilar/mediastinal lymphadenopathy, consolidation, cavity (solitary and multiple), infiltrate, fibrosis, calcification, atelectasis, pleural effusion, bronchopneumonic changes, miliary opacification, pleural thickness, bronchiectasis, pneumothorax, and giant bullae.

TB severity was assessed using the Classification of TB Severity by Wiseman et al. based on lesion appearance in CXR [9]. Expansile alveolar opacification, multilobar alveolar opacification, tuberculous bronchopneumonia, cavitation, complicated intrathoracic lymph node disease, and empyema are classified as severe disease.

### 2.4 Statistics

Data were analyzed statistically using IBM SPSS Statistics version 25.0 and Microsoft Excel 2019. The CXR images of DR‐TB and DS‐TB and the severity of tuberculosis were compared using Pearson chi‐square and Fisher's exact test. A *p*‐value less than 0.05 was considered significant.

## 3 Results

### 3.1 Characteristics

Sixty‐nine children, 31 with DR‐TB and 38 with DS‐TB were assessed. The female sex was preponderant in both diagnoses (65% DR‐TB vs. 82% DS‐TB), and most children were aged 16–18 years (52% DR‐TB vs. 24% DS‐TB). More than half of the children underwent a CXR before treatment started (87% DR‐TB vs. 63% DS‐TB); thus, this was used as the baseline. Most children with DR‐TB (74%) had no previous drug treatment history, but previous drug treatment history was unknown in 61% of the children with DS‐TB, probably because the previous drug treatment history is not asked in anamnesis or not recorded in their medical status.

Table 1 presents the pulmonary DR‐TB and DS‐TB characteristics in children, showing that the TB diagnosis was confirmed bacteriologically in all DR‐TB cases (100%), while more than half of the DS‐TB cases (53%) were clinically diagnosed. Of the 31 children diagnosed with DR‐TB, 65% were classified as MDR‐TB, followed by 19% of RR‐TB, 13% of pre‐XDR‐TB, and 3% of XDR‐TB. None of the children with DR‐TB were HIV positive, whereas four children with DS‐TB were HIV positive. Most of the children with DS‐TB had unknown HIV status, probably because they were not tested or it was not recorded in their medical status.

**TABLE 1:** Characteristics of children by pulmonary DR‐TB and pulmonary DS‐TB (*n* = 69).

Characteristics	DR‐TB, *n* (%)	DS‐TB, *n* (%)
Total patients	31 (45)	38 (55)
Sex		
Male	11 (36)	7 (18)
Female	20 (65)	31 (82)
Age (years)		
0–5	2 (7)	9 (24)
6–10	2 (7)	6 (16)
11–15	11 (36)	14 (37)
16–18	16 (52)	9 (24)
Drug treatment status		
Before treatment	27 (87)	24 (63)
During treatment	4 (13)	14 (37)
Drug treatment history		
Present	7 (23)	13 (34)
Not present	23 (74)	2 (5)
Unknown	1 (3)	23 (61)
TB diagnosis		
Bacteriologically confirmed	31 (100)	18 (47)
Clinically diagnosed	0 (0)	20 (53)
Types of drug resistance		
RR‐TB	6 (19)	
MDR‐TB	20 (65)	
Pre‐XDR‐TB	4 (13)	
XDR‐TB	1 (3)	
HIV status		
Positive	0 (0)	4 (11)
Negative	28 (90)	11 (30)
Unknown	3 (10)	23 (61)

### 3.2 Chest X‐Ray Findings

**FIGURE 1:**
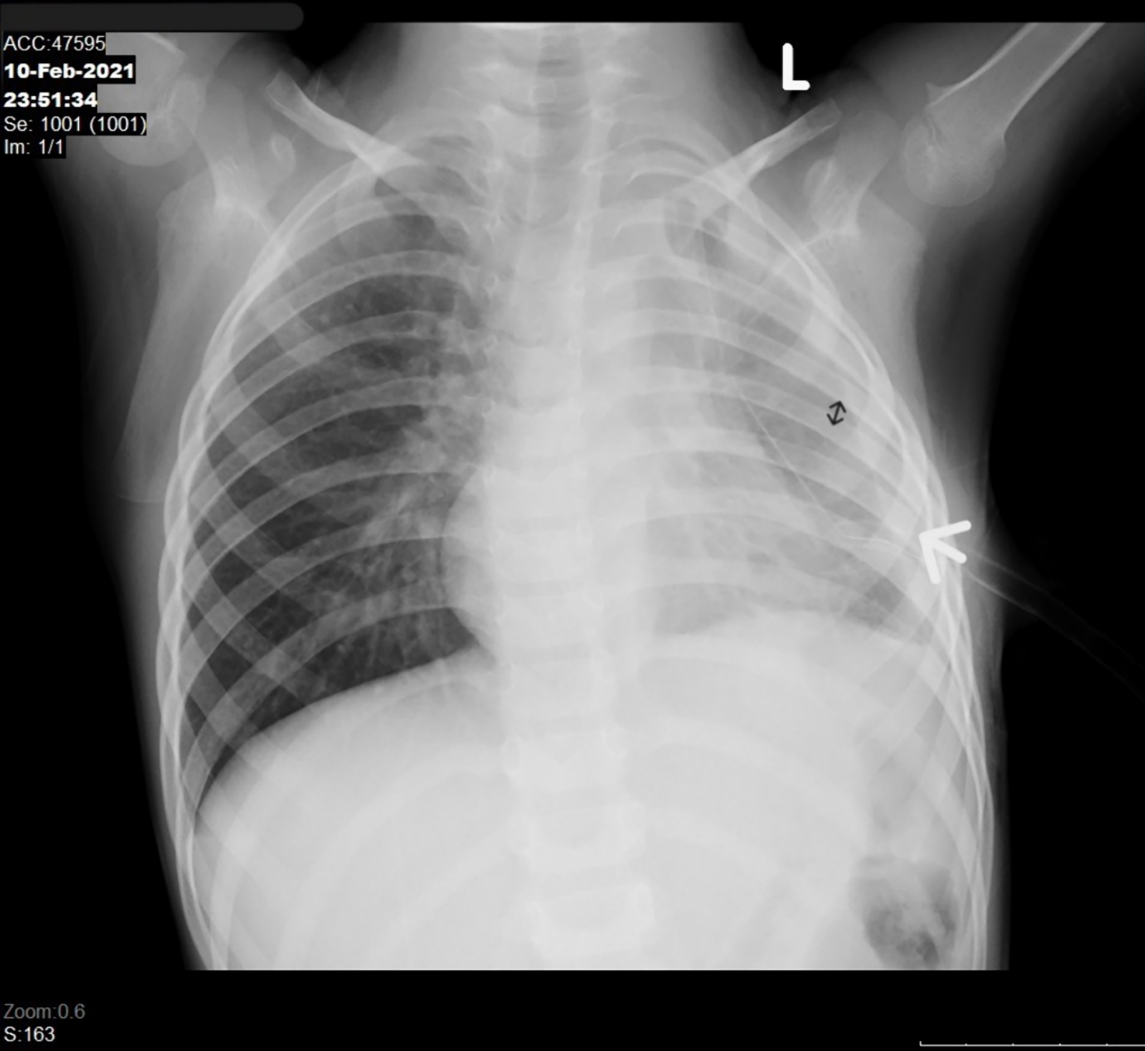
Pulmonary DS‐TB in 5‐year‐old boy. CXR was taken in AP projection with findings of left pleural effusion (white arrow) accompanied by narrowing of the intercostal space (black arrow). There is enlargement of right perihilar lymph node. AP, anteroposterior; CXR, chest X‐ray; DS‐TB, drug‐sensitive TB.

**FIGURE 2:**
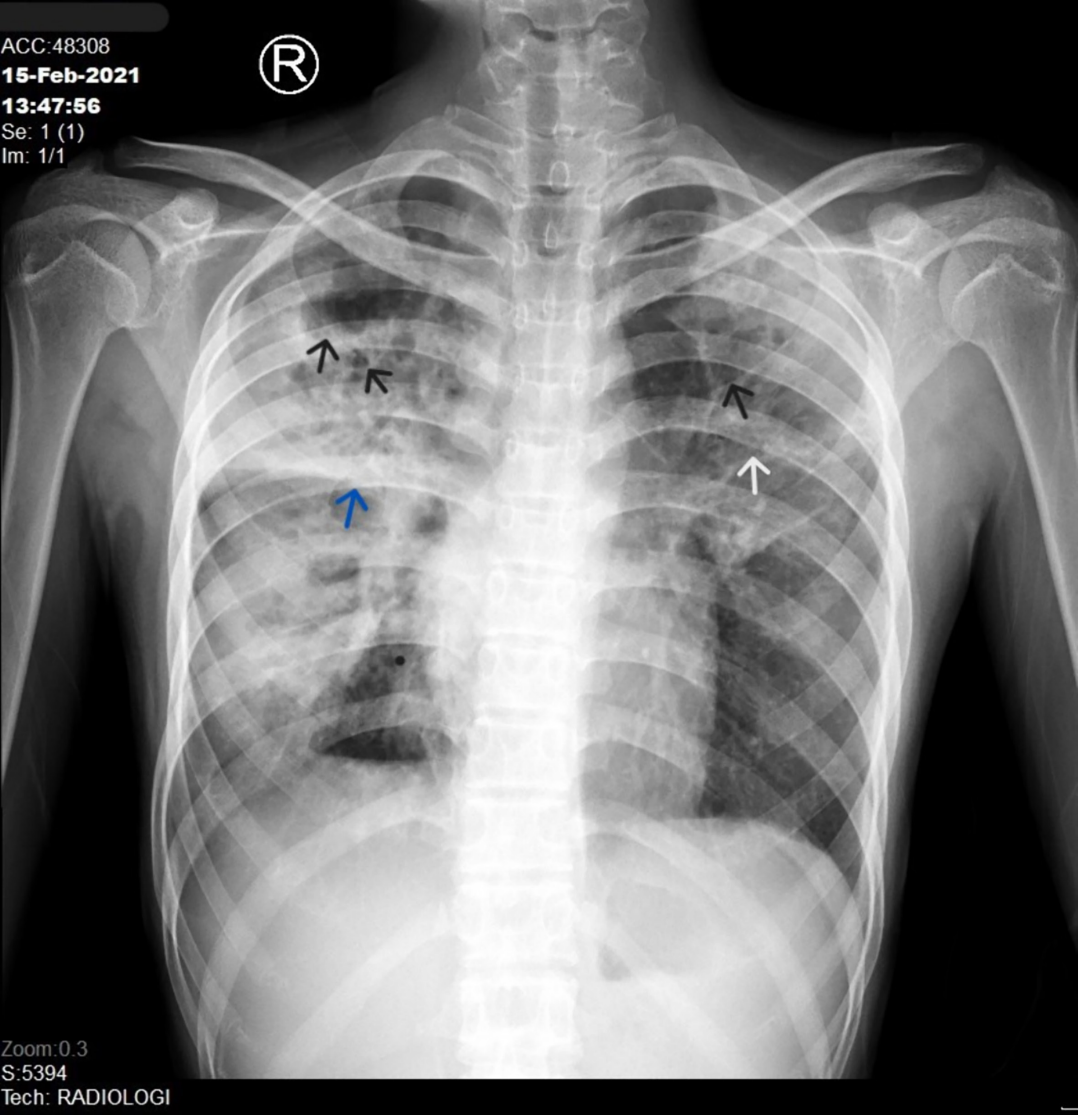
Pulmonary DR‐TB in 16‐year‐old girl. CXR was taken in AP projection with findings of multiple cavities (black arrows) in the bilateral upper zone of lungs and middle zone of left lung. There were also fibrosis (white arrow) and consolidation in the both lungs. Minor fissure thickness (blue arrow) was also seen. AP, anteroposterior; CXR, chest X‐ray; DR‐TB, drug‐resistant TB.

As shown in Table 2, three children with DR‐TB and five children with DS‐TB had a normal CXR. The most common finding in children with DR‐TB was consolidation (68% DR‐TB vs. 18% DS‐TB, *p* < 0.001), while it was pleural effusion in children with DS‐TB (10% DR‐TB vs. 37% DS‐TB, *p* = 0.009) (Figure 1). The cavity (29% DR‐TB vs. 5% DS‐TB, *p* = 0.009) and fibrosis (42% DR‐TB vs. 13% DS‐TB, *p* = 0.007) were also found mainly in DR‐TB children (Figure 2). More than half of the cavities found in DR‐TB were multiple (*n* = 8, 89%), but there was no significant difference in the number of cavities between DR‐TB and DS‐TB (*p* = 0.345).

**TABLE 2:** Chest X‐ray comparison of pulmonary DR‐TB and pulmonary DS‐TB in children.

CXR results (*n* = 69)	DR‐TB, *n* (%)	DS‐TB, *n* (%)	*p*
Total patients	31 (45)	38 (55)	
Normal	3 (10)	5 (13)	0.722
Hilar/mediastinal lymphadenopathy	2 (7)	7 (18)	0.171
Consolidation	21 (68)	7 (18)	<0.001
Cavity	9 (29)	2 (5)	0.009
Solitary	1 (11)	1 (50)	0.345
Multiple	8 (89)	1 (50)	
Infiltrate	6 (19)	5 (13)	0.525
Fibrosis	13 (42)	5 (13)	0.007
Calcification	7 (23)	2 (5)	0.068
Atelectasis	1 (3)	1 (3)	1.000
Pleural effusion	3 (10)	14 (37)	0.009
Bronchopneumonic changes	1 (3)	0 (0)	0.449
Miliary opacification	1 (3)	0 (0)	0.449
Pleural thickness	5 (16)	1 (3)	0.083
Bronchiectasis	0 (0)	1 (3)	1.000
Pneumothorax	1 (3)	2 (5)	1.000
Giant bullae	1 (3)	0 (0)	0.449

Hilar/mediastinal lymphadenopathy, infiltrate, calcification, atelectasis, bronchopneumonic changes, miliary opacification, pleural thickness, bronchiectasis, pneumothorax, and giant bullae were also observed, but there were no significant differences between the groups.

### 3.3 Severity

Table 3 shows a significant difference in TB severity between children with DR‐TB and DS‐TB, with half of the children with DR‐TB classified as severe TB (50% DR‐TB vs. 19% DS‐TB, *p* = 0.008). Some severity could not be assessed according to existing criteria in four children due to incomplete CXR interpretation of the lesion size.

**TABLE 3:** TB severity of pulmonary DR‐TB and pulmonary DS‐TB in children.

TB severity	DR‐TB, *n* (%)	DS‐TB, *n* (%)	*p*
Total patients	28 (43)	37 (57)	
TB severity (*n* = 65)			0.008
Severe TB	14 (50)	7 (19)	
Non‐severe TB	14 (50)	30 (81)	

## 4 Discussion

There are only a few studies of DR‐TB in children globally, especially regarding comparisons between pulmonary DR‐TB and DS‐TB. The number of females in the present study was in line with studies by Lapphra et al., Seddon et al., and Seddon et al. for DR‐TB only, where there were more female cases than males [4, 10, 11]. The higher number of children diagnosed in the age group ≥15 years was in line with studies in Korea and Pakistan [12, 13]. These results suggest a much greater likelihood of recurrent TB and the risk of developing adult type TB [13, 14]. However, this highlights the possibility of younger children being “underdiagnosed” while having the same potential for developing TB and its resistance as in older age group [15].

MDR‐TB was the most common resistance type found in the present study, followed by, in descending pattern, RR‐TB, pre‐XDR‐TB, and XDR‐TB, in line with previous studies by Seddon et al., Kim et al., and Malik et al. [10, 12, 13] However, the HIV status differed from a previous study in which HIV co‐infection was observed to be higher in DR‐TB than DS‐TB [4]. Based on previous studies, there is no significant association of positive‐HIV status among DR‐TB patients, either in children or in adults [4, 16, 17, 18]. However, our study had 26 children with unknown HIV, which may affect the overall analysis.

Consolidation is related to parenchymal granulomatous inflammation and suggestive of primary and postprimary tuberculosis [1]. The highest proportion of consolidation was observed in CXR findings of children with DR‐TB, consistent with the studies by Manikkam et al. and Seddon et al., which reported that over half of children had segmental or lobar consolidation [5, 10]. Consolidation in DR‐TB was also included as one of the two findings with the highest proportion in a previous study compared with DS‐TB. However, it was independently associated after thorough multivariate analysis [4].

Consolidation was more dominant in DR‐TB, as reported in a previous study in adults, and with cavities, it is probably caused by recurrent TB initiated by failure in treatment or *Mycobacterium tuberculosis* (MTB) mutation [19]. Children with MDR‐TB who had previously undergone TB treatment also show higher consolidation and more cavities than children without a history of TB treatment [5]. It was also reflected in our study where all children with DR‐TB who had previous drug treatment history had consolidation as findings in their CXR. Thus, the history of previous TB or previous anti‐TB treatment might be a risk to the development of consolidation in children with DR‐TB.

A study of adults with MDR‐TB in Indonesia reported that cavity, infiltrate, nodule, and fibrosis were significantly associated with MDR‐TB incidence [20]. Bronchopneumonic changes, described as a nodule in the same Indonesia study, is the only finding that can be used to predict the presence of MDR‐TB after multivariate analysis [20]. This differed from our study, where bronchopneumonic changes were only found in one child with DR‐TB and were not statistically significant.

However, there was a high proportion of fibrosis in our study that was significantly associated with DR‐TB. Fibrosis and calcification are lesions of the “recovered” granuloma and usually indicate previous or inactive tuberculosis [1, 21]. Fibrosis was observed in most of the children in our DR‐TB group who had a history of previous drug treatment.

Another parenchymal damage found higher in proportion in DR‐TB is the cavity. It was one of the findings with the highest frequency in three previous studies [5, 10, 11]. In our study, the number of cavities was mainly multiple in a DR‐TB, but there was no significant difference. This is in line with a study of adults that reported more cavities in DR‐TB than DS‐TB with other characteristics of maximum diameter ≥30 mm, number of cavities ≥3, and involvement ≥2 lungs zone [16]. Another adult study reported cavity as the predominant CXR findings in DR‐TB with non‐reactive HIV, especially multiple cavities [22]. The cavity is formed when there is a fusion between necrotic granuloma and the bronchus. Oxygen presence in the cavity supports the bacteria proliferation and, along with the wall thickness of the cavity, may be involved in the mechanism of DR‐TB [21, 23].

In our study, there was more pleural effusion in DS‐TB than in DR‐TB, which differs from a study in Thailand that found the highest frequency of pleural effusion in DR‐TB [4]. Previous adult studies also reported a higher proportion of pleural effusion in DR‐TB [16]. However, a study of adults in Uganda reported similar results whereby there was more pleural effusion in DS‐TB than DR‐TB in HIV‐negative adults [23]. Most of the children in the present study diagnosed with DS‐TB and pleural effusion were from inpatient care and thus assumed to have higher bacillary loads resulting in more severe and complicated forms of TB [25]. Those bacterial load in DR‐TB patients resulting in the excessive entry of MTB into the pleural space [16]. Discharged bacteria to the pleura space caused by pulmonary focus or caseated lymph nodes in the subpleural results in pleura effusion [24].

There was a significant difference in TB severity between DR‐TB and DS‐TB, with more severe TB in the DR‐TB group. Classification is based on criteria proposed by Wiseman et al., where children with severe TB mainly had a cavity [9]. Furthermore, eight children had multilobar alveolar opacification, one had expansile alveolar opacification, two had atelectasis, and one had empyema. Pneumothorax found in three children in this study was classified as severe TB as there is a probability of it being a complication from the ruptured cavity [26].

Previous adult studies reported that DR‐TB had more severe lung parenchymal damage than DS‐TB [16, 20]. TB severity is associated with culture‐convert, treatment outcomes, and mortality in the study of children with DR‐TB. While non‐severe TB has better treatment outcomes, adjusted analysis suggests a better prognosis can be achieved in children with MDR‐TB who are identified and treated appropriately [10, 11].

This study has several limitations. First, it may be underpowered as the minimum number of children with DR‐TB was not analyzed. Most DS‐TB data were also collected from in‐patient medical records, which may have more complicated manifestations. Not all children with DS‐TB were bacteriologically confirmed; thus, there might be underestimated drug resistance. Our study did not include other comorbidities than HIV, such as diabetes mellitus, where radiological manifestation might be different or be more severe than in children without comorbidities. Furthermore, children with unknown HIV status will be subject to bias in assessing the severity of radiological manifestations.

The CXR findings were evaluated by the same radiologist, a pediatric radiology consultant, thus minimizing the framing bias, but there is the possibility of missed findings. Few lateral projections of CXR were also a study limitation as it is essential to detect some TB findings, especially mediastinal or hilar lymphadenopathy. However, our study is still one of few studies of CXR findings comparing children with pulmonary DR‐TB and pulmonary DS‐TB. The similarity of CXR findings in children to adults may indicate a similarity in TB progression to the existing radiological manifestations, such as consolidation, which were related to treatment history. This becomes a concern because TB in children is often underdiagnosed, but their disease progression is similar to that experienced by adults. Therefore, it is necessary to promote further diagnosis and treatment of TB in children.

In conclusion, the most common findings in CXR are consolidation, fibrosis, and cavities in children with pulmonary DR‐TB and pleural effusion in children with pulmonary DS‐TB. The proportion of severe TB is also higher in children with pulmonary DR‐TB than pulmonary DS‐TB. These findings can potentially be considered in further examination of children with pulmonary DR‐TB and DS‐TB, but further extensive studies with more children in various settings are required to confirm these results.

## Author Contributions

**Saffanah Az Zuhriyyah:** methodology, investigation, formal analysis, data curation, writing – original draft, writing – review and editing, visualization. **Harry Galuh Nugraha:** validation, investigation, resources, data curation, writing – original draft, writing – review and editing, visualization, supervision. **Djatnika Setiabudi:** methodology, validation, writing – review and editing, supervision. **Prayudi Santoso:** methodology, validation, writing – review and editing, supervision. **Heda Melinda Nataprawira:** conceptualization, methodology, validation, formal analysis, investigation, resources, data curation, writing – original draft, writing – review and editing, visualization, supervision, project administration.

## Ethics Statement

This study was conducted after obtaining an ethical exemption letter from Universitas Padjadjaran's Research Ethics Committee, number 862/UN6.KEP/EC/2022 and research permit letter from the director of Dr. Hasan Sadikin General Hospital Bandung, number LB.02.01/X.2.2.1/20936/2022. Individual informed consent was waived due to no direct interaction with the patient, and only medical record was used during this study.

## Conflicts of Interest

The authors declare no conflicts of interest.

## Data Availability

Research data are not shared.
